# Supplementary material for: Composition Effect on the Formation of Oxide Phases by Thermal Decomposition of CuNiM(III) Layered Double Hydroxides with M(III) = Al, Fe
Source: Materials (Basel). 2023 Dec 23;17(1):83. doi: 10.3390/ma17010083 (PMC10779612; doi:10.3390/ma17010083)
Supplement: Supplementary file 1 [file materials-17-00083-s001.zip › materials-2758460-supplementary.pdf]

SUPPORTING INFORMATION

Composition effect on the formation of oxide phases by thermal decomposition of CuNiM(III) layered double hydroxides with M(III) = Al, Fe

Iqra Zubair Awan[1,2,3], Phuoc Hoang Ho[1,4], Giada Beltrami[5], Bernard Fraisse[1], Thomas Cacciaguerra[1], Pierrick Gaudin[1], Nathalie Tanchoux[1], Stefania Albonetti[2], Annalisa Martucci[5], Fabrizio Cavani[2], Francesco Di Renzo[1], Didier Tichit[1,] *

[1]ICGM, Université de Montpellier-CNRS-ENSCM, Montpellier, France

[2]Dip. Chimica Industriale Toso Montanari, Alma Mater Studiorum Università di Bologna,

Bologna, Italy

[3]Dpt Chemistry, Lahore Garrison University, Lahore, Pakistan

[4]Chemical Engineering, Competence Centre for Catalysis, Chalmers University of Technology, SE-412 96, Gothenburg, Sweden

[5]Dpt Physics and Earth Sciences, University of Ferrara, Ferrara, Italy

Table S1 : Mass % and Scherrer crystallite size of phases at different temperatures in the XRD thermal ramp of (Cu,Ni)Al samples

Mass %

**T / °C**	**Ni75Al25**			**Cu07Ni68Al25**			**Cu38Ni37Al25**			**Cu68Ni07Al25**			**Cu75Al25**		
	**CuO**	**NiO**	**spinel**	**CuO**	**NiO**	**spinel**	**CuO**	**NiO**	**spinel**	**CuO**	**NiO**	**spinel**	**CuO**	**NiO**	**spinel**
300	-	40.1	-	-	47.8	-	3.5	8.1	-	24.6	-	-	11.8	-	-
400	-	44.1	-	-	50.4	-	3.5	7.5	-	29.7	-	-	11.3	-	-
500	-	47.4	-	-	52.6	-	6.9	18.0	-	29.8	-	-	11.8	-	-
600	-	53.3	-	-	49.4	-	8.2	21.3	-	36.4	-	-	35.9	-	-
700	-	55.6	-	-	50.9	-	19.3	25.9	-	44.0	-	-	43.7	-	11.3
800	-	57.5	-	-	52.6	-	29.7	32.8	-	50.5	-	19.4	62.2	-	36.6

Scherrer crystallite size / nm

**T / °C**	**Ni75iAl25**			**Cu07Ni68Al25**			**Cu38Ni37Al25**			**Cu68Ni07Al25**			**Cu75Al25**		
	**CuO**	**NiO**	**spinel**	**CuO**	**NiO**	**spinel**	**CuO**	**NiO**	**spinel**	**CuO**	**NiO**	**spinel**	**CuO**	**NiO**	**spinel**
300	-	2.5	-	-	2.1	-	-	-	-	8.4	-	-	7.7	-	-
400	-	2.5	-	-	2.2	-	-	-	-	8.9	-	-	8.0	-	-
500	-	2.7	-	-	2.6	-	10.5	4.7	-	9.4	-	-	7.7	-	-
600	-	3.2	-	-	2.8	-	12.0	4.4	-	10.5	-	-	10.5	-	-
700	-	3.4	-	-	3.4	-	15.3	7.4	-	13.6	-	-	12.1	-	11.5
800	-	4.0	-	-	3.9	-	25.5	11.4	-	19.6	-	9.6	16.2	-	12.3

Table S2 : Mass % and Scherrer crystallite size of phases at different temperatures in the XRD thermal ramp of (Cu,Ni)Fe samples

**T / °C**	**Ni75Fe25**			**Cu07Ni68Fe25**			**Cu38Ni37Fe25**			**Cu68Ni07Fe25**			**Cu75Fe25**		
**Mass %**															
	**CuO**	**NiO**	**spinel**	**CuO**	**NiO**	**spinel**	**CuO**	**NiO**	**spinel**	**CuO**	**NiO**	**spinel**	**CuO**	**NiO**	**spinel**
300	-	58.2	-	-	25.6	-	15.1	21.6	-	46.0	-	-	47.7	-	-
400	-	65.4	-	-	30.7	-	18.8	31.9	-	44.8	-	-	50.4	-	-
500	-	61.1	-	-	31.6	-	18.8	36.3	-	47.7	-	-	52.7	-	-
600	-	52.0	-	-	29.8	12.8	20.9	40.1	16.9	40.8	2.6	11.1	51.9	-	18.7
700	-	55.6	3.8	-	43.9	20.8	32.6	38.5	25.6	55.7	4.3	31.2	57.0	-	25.6
800	-	44.9	12.3	-	63.5	36.5	27.1	35.3	37.6	54.1	4.0	27.3	56.9	-	25.3
**Scherrer crystallite size / nm**															
**T / °C**	**Ni75Fe25**			**Cu07Ni68Fe25**			**Cu38Ni37Fe25**			**Cu68Ni07Fe25**			**Cu75Fe25**		
	**CuO**	**NiO**	**spinel**	**CuO**	**NiO**	**spinel**	**CuO**	**NiO**	**spinel**	**CuO**	**NiO**	**spinel**	**CuO**	**NiO**	**spinel**
300	-	2.1	-	-	2.4	-	7.0	2.4	-	50.7			6.0	-	-
400	-	2.4	-	-	2.8	-	6.0	3.4	-	49.3			6.0	-	-
500	-	3.1	-	-	3.6	-	6.0	3.6	-	52.5			5.6	-	-
600	-	3.9	-	-	5.0	8.2	9.4	5.7	9.1	44.9		12.2	8.4	-	6.9
700	-	4.7	7.5	-	8.1	7.5	16.8	9.3	13.7	61.4		34.3	16.9	-	12.7
800	-	7.4	13.7	-	18.3	16.5	25.5	12.6	16.5	59.4		30.0	32.5	-	25.7

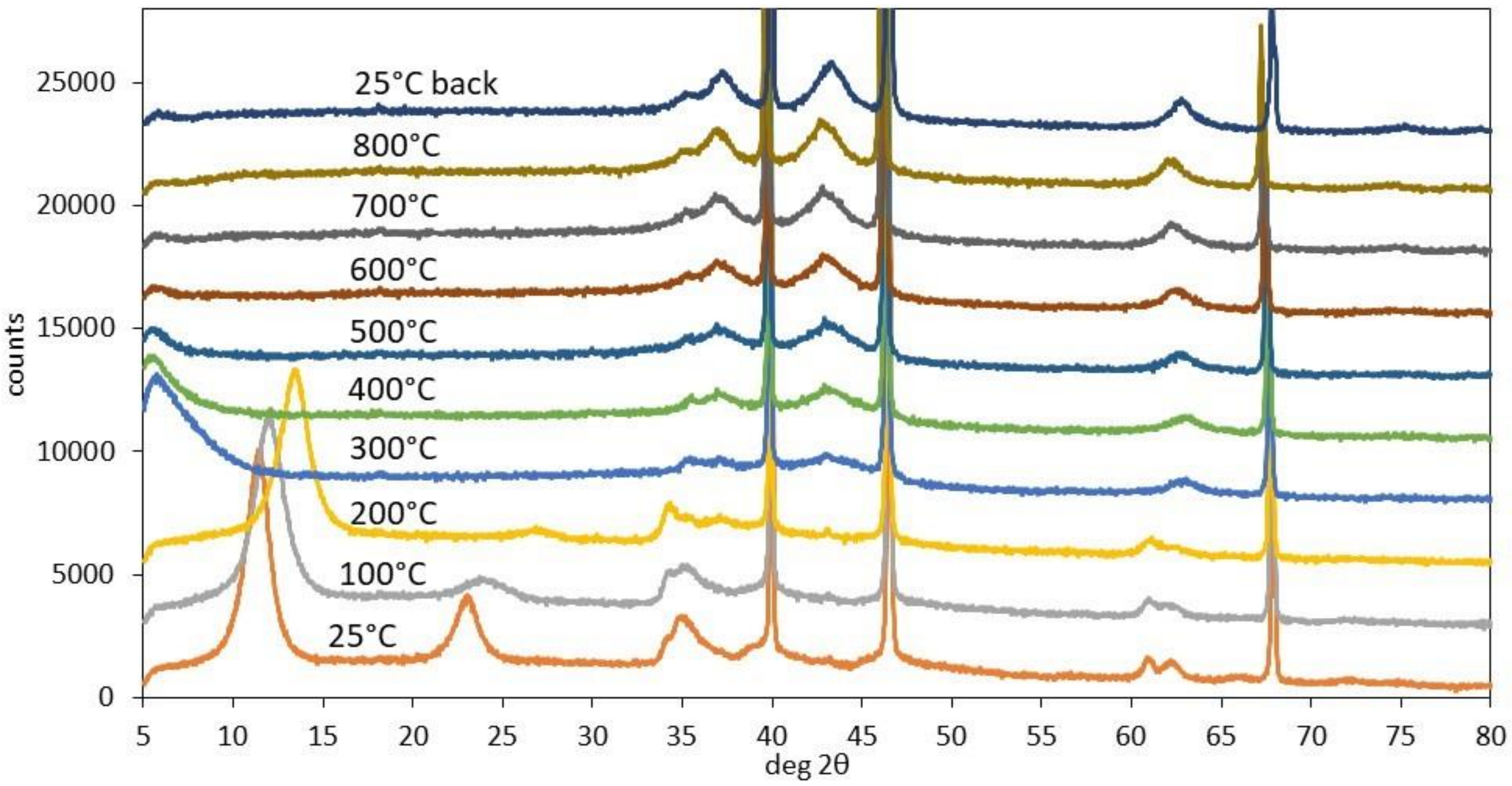

Figure S1. XRD patterns of Ni75Al25: temperature ramp from room temperature to 800°C and back.

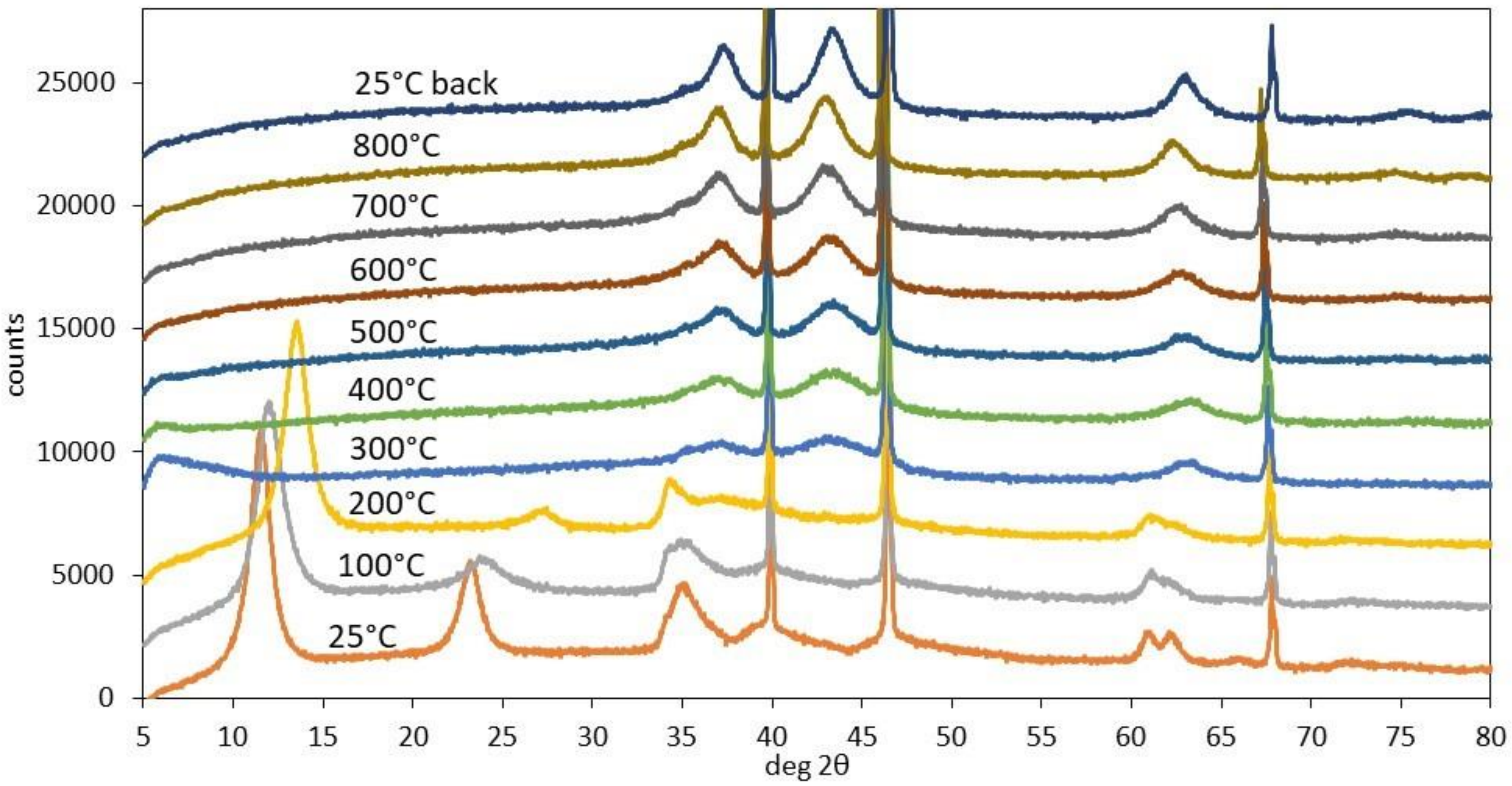

Figure S2. XRD patterns of Cu07Ni68Al25: temperature ramp from room temperature to 800°C and back.

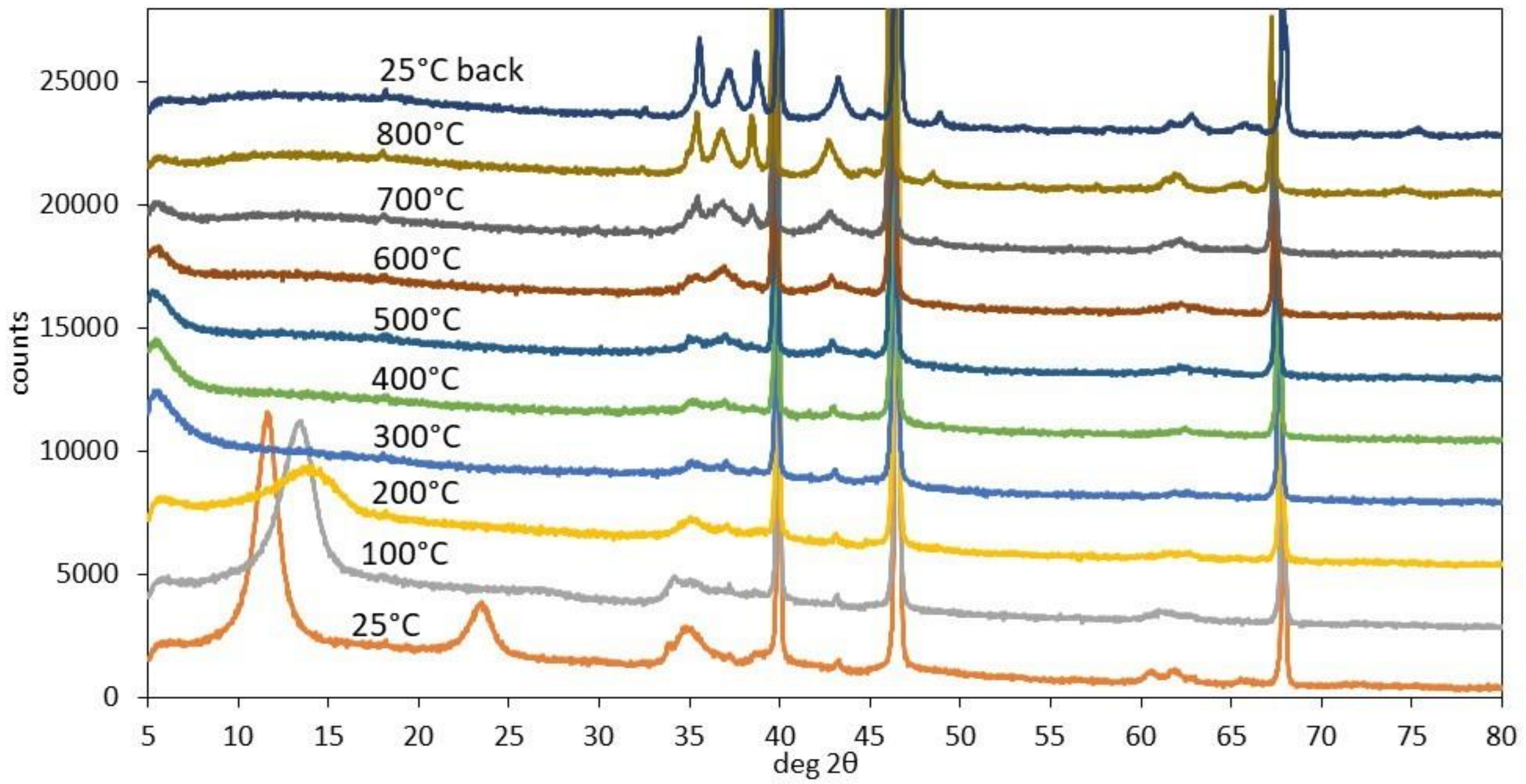

Figure S3. XRD patterns of Cu38Ni37Al25: temperature ramp from room temperature to 800 °C and back.

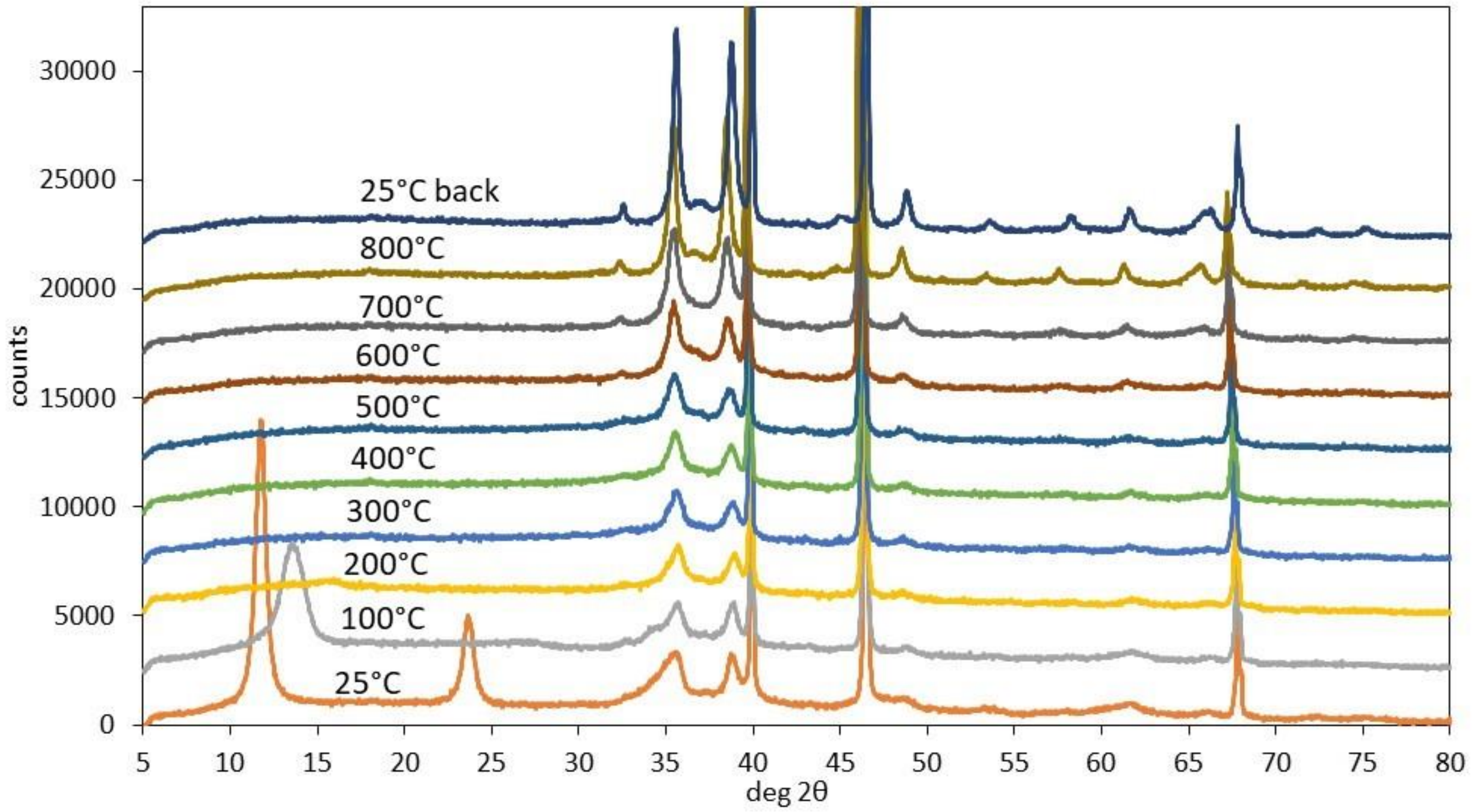

Figure S4. XRD patterns of Cu68Ni07Al25: temperature ramp from room temperature to 800 °C and back.

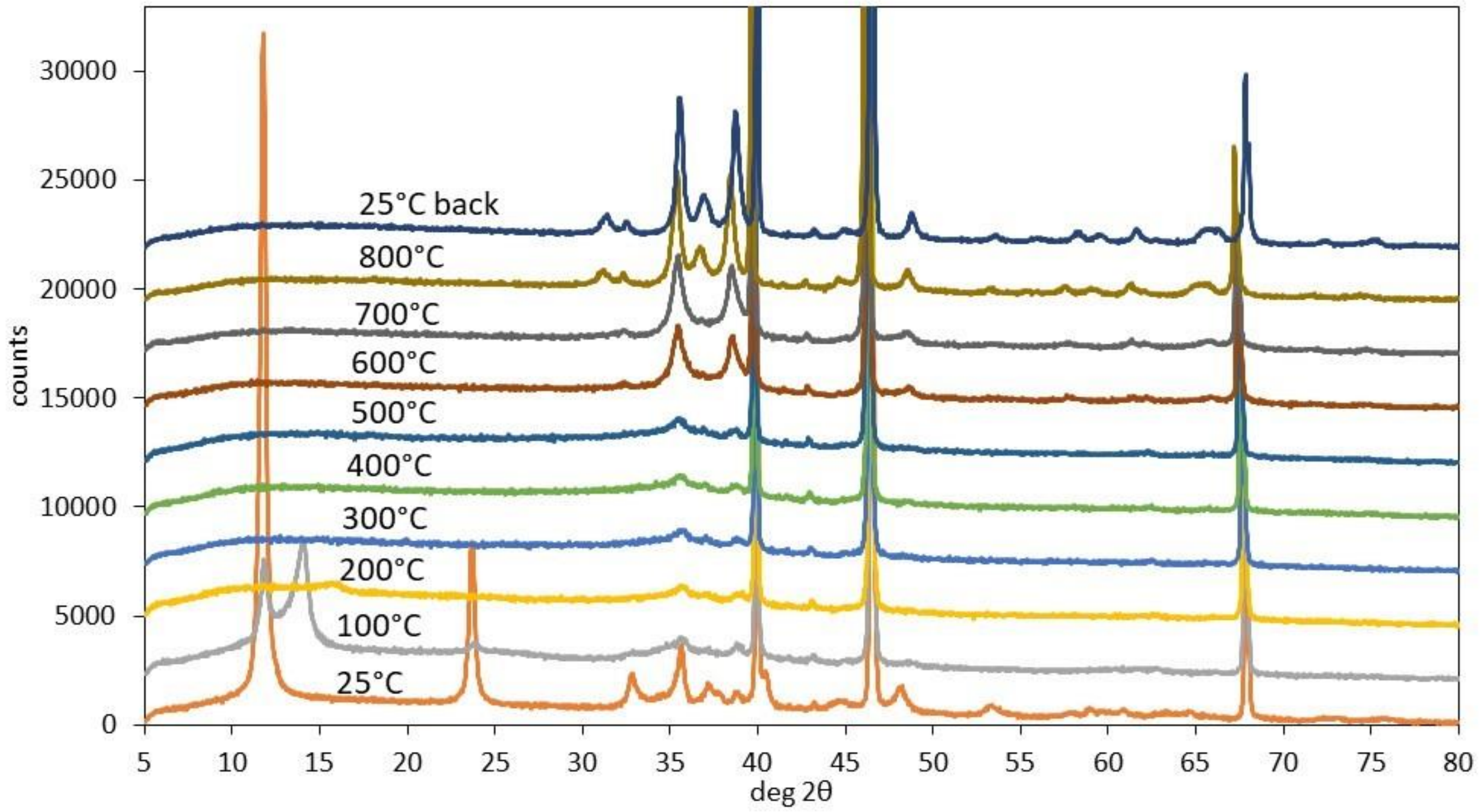

Figure S5. XRD patterns of Cu75Al25: temperature ramp from room temperature to 800 °C and back.

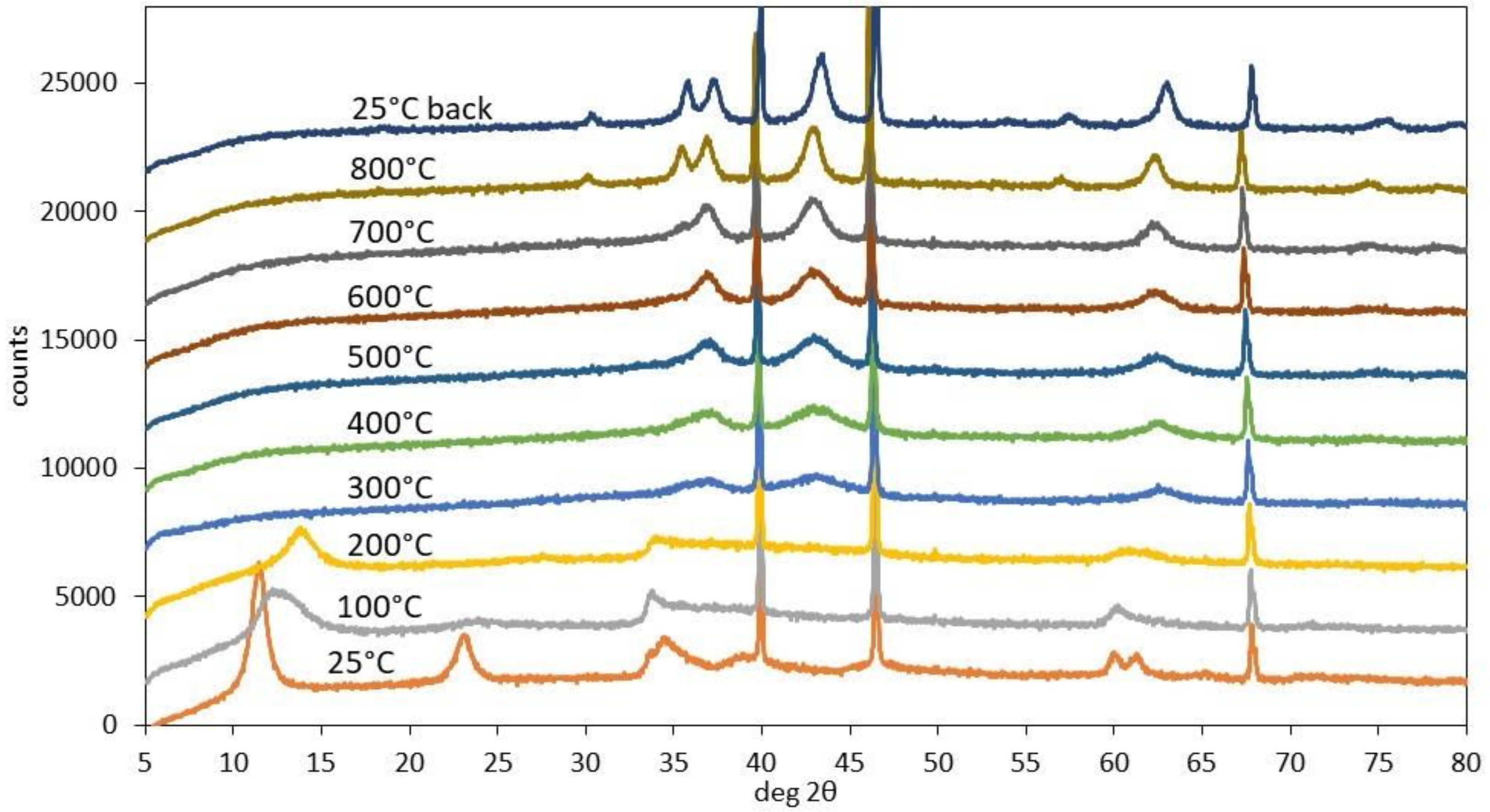

Figure S6. XRD patterns of Ni75Fe25: temperature ramp from room temperature to 800 °C and back.

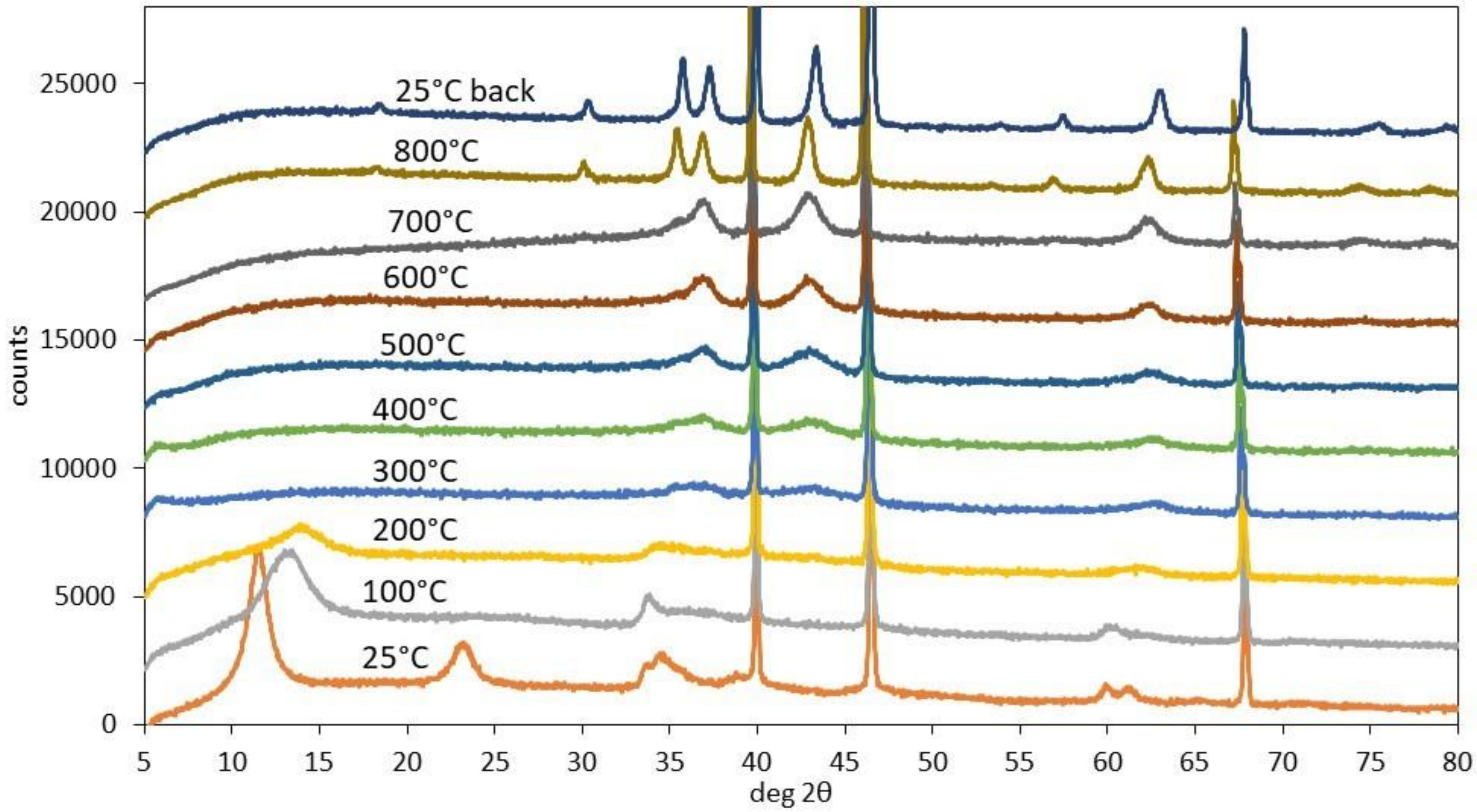

Figure S7. XRD patterns of Cu07Ni68Fe25: temperature ramp from room temperature to 800 °C and back.

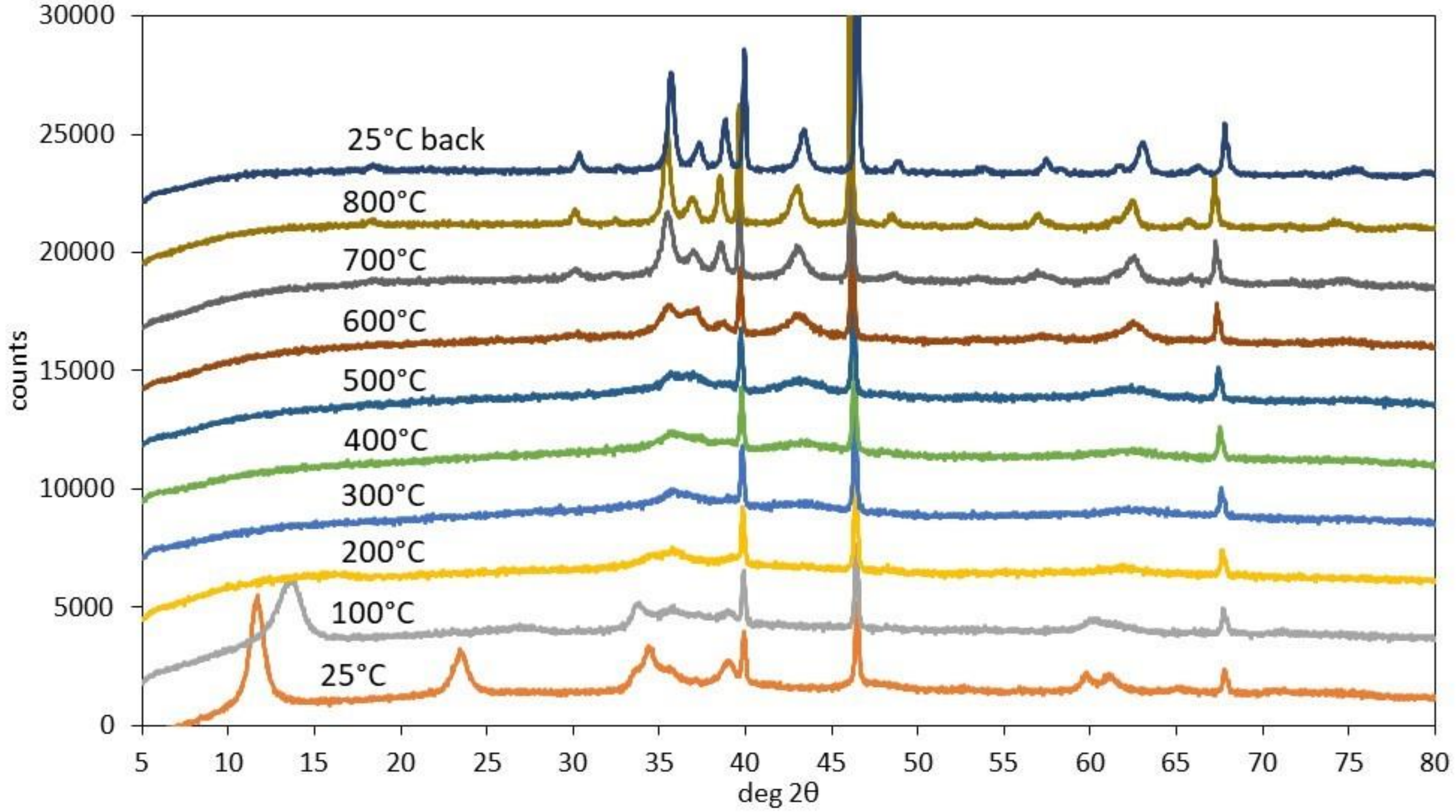

Figure S8. XRD patterns of Cu38Ni37Fe25: temperature ramp from room temperature to 800 °C and back.

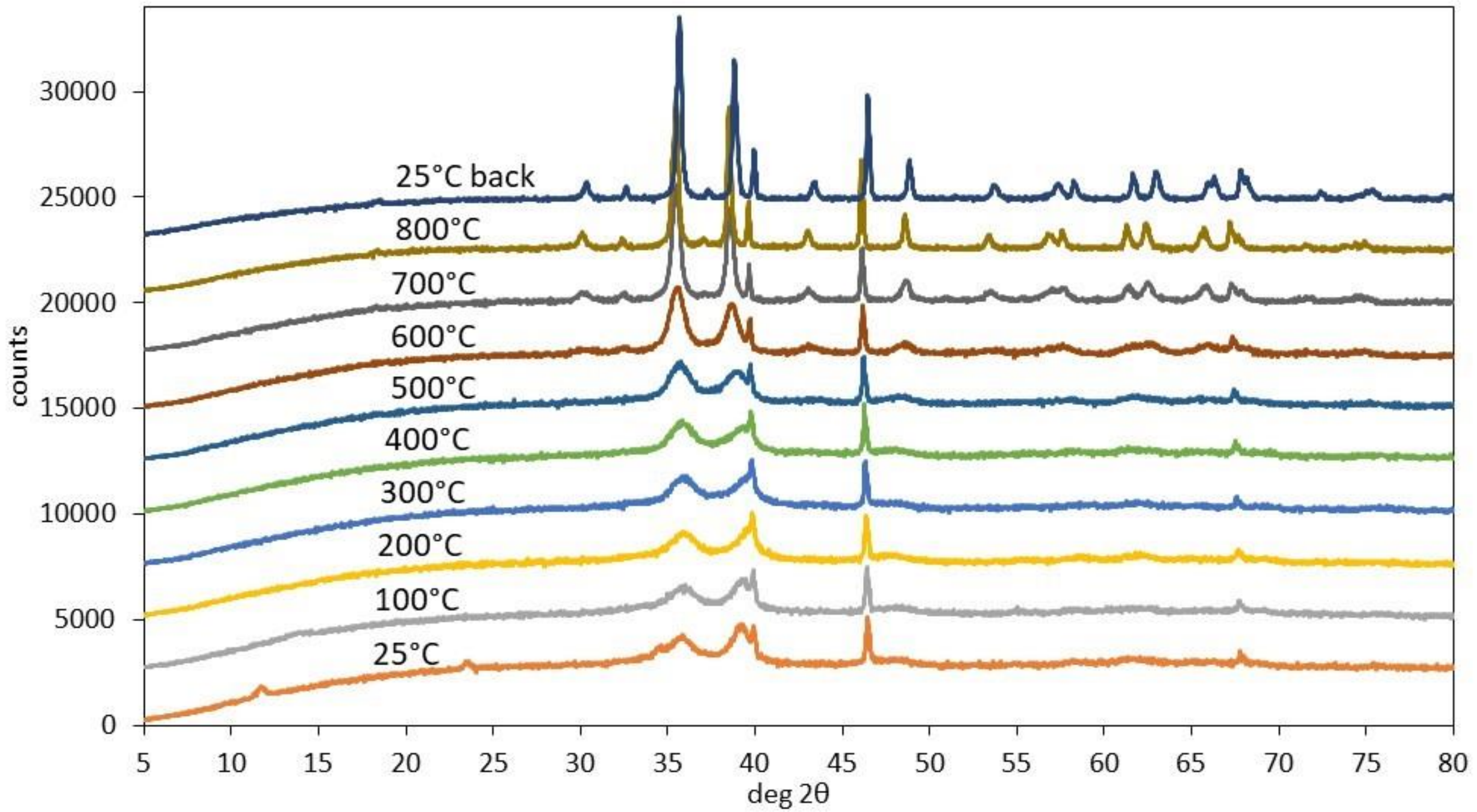

Figure S9. XRD patterns of Cu68Ni07Fe25: temperature ramp from room temperature to 800 °C and back.

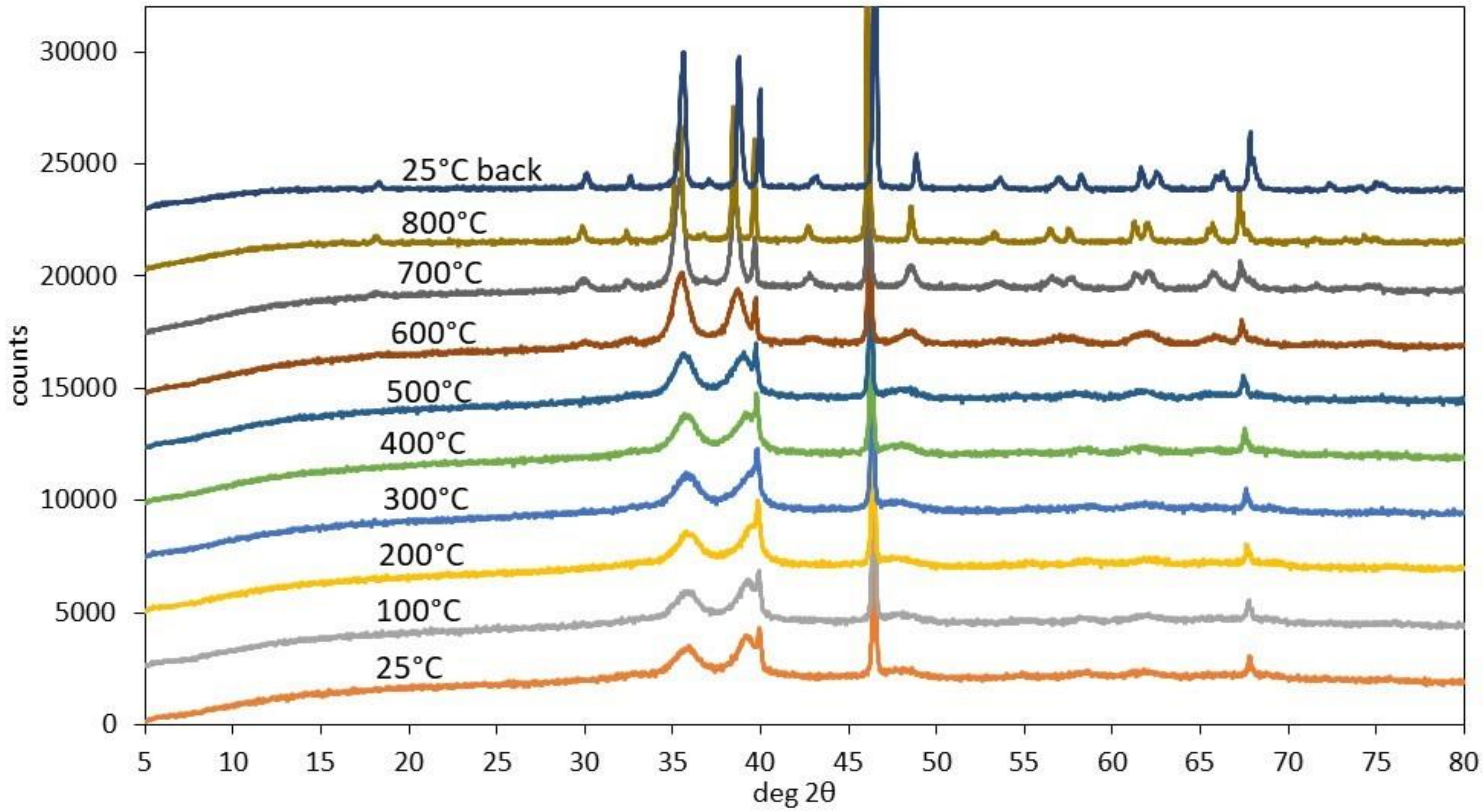

Figure S10. XRD patterns of Cu75Fe25: temperature ramp from room temperature to 800 °C and back.
